# Sicca Symptoms, Oral Health Conditions, Salivary Flow and Oral *Candida* in Sjögren’s Syndrome Patients

**DOI:** 10.3390/ijerph17103625

**Published:** 2020-05-21

**Authors:** Weini Xin, Katherine Chiu Man Leung, Edward Chin Man Lo, Mo Yin Mok, Moon Ho Leung

**Affiliations:** 1 Department of Stomatology, Shantou University Medical College, Shantou 515041, China; wnxin@stu.edu.cn; 2 Faculty of Dentistry, The University of Hong Kong, Pok Fu Lam, Hong Kong; hrdplcm@hku.hk; 3 Department of Biomedical Sciences, City University of Hong Kong, Kowloon, Hong Kong; temy.mok@cityu.edu.hk; 4 Department of Medicine, Queen Elizabeth Hospital, Hong Kong SAR, Hong Kong; leungmh@ha.org.hk

**Keywords:** Sjögren’s syndrome, Sicca Symptoms Inventory, patient-reported outcomes, oral *Candida*

## Abstract

This study aimed to investigate the relationships among sicca symptoms, oral health conditions, salivary profiles and oral Candida in Sjögren’s syndrome (SS) patients. Eighty-five SS patients (mean age = 50.5) and 40 healthy non-SS individuals (mean age = 51.4) were recruited. They self-completed the Sicca Symptoms Inventory (SSI). Decayed, missing and filled surface (DMFS) scores, salivary flow rates, pH and oral Candida colonization were determined. Mean SSI summary scores of SS patients and non-SS individuals were 11.1 and 5.4 respectively (*p* < 0.001). The most prevalent sicca symptoms in SS patients were eye irritation (93%), dry throat or nose (88%) and need of fluid for mouth wetting (88%). SS patients had significantly lower whole salivary flow rates than the non-SS individuals. Candida strains were isolated from over 60% of SS patients but not in non-SS patients. C. albicans was the predominant species. SSI summary score was negatively correlated to salivary flow rates while SSI summary and domain scores were positively correlated to the number of filled surfaces (FS) and DMFS scores and oral Candida counts. In conclusion, SS patients had more severe sicca symptoms than non-SS individuals. SSI scores were negatively correlated to the salivary flow rates but positively correlated to caries experience and oral Candida colonization.

## 1. Introduction

Sjögren’s syndrome (SS) is a slowly progressive autoimmune disorder of exocrine glands which is characterized by intense lymphocytic infiltration [1]. Primary SS (pSS) occurs in the absence of connective tissue diseases and secondary Sjögren’s syndrome (sSS) is associated with connective tissue diseases such as rheumatoid arthritis (RA), systemic lupus erythematosus (SLE) or systemic sclerosis [2,3]. Destruction of the exocrine gland tissues results in reduction of salivary, lacrimal and other exocrine secretions. Dry mouth and dry eyes are major chief complaints of patients with SS. SS sufferers may present with other sicca symptoms such as skin and vaginal dryness, as well as fatigue and somatic discomfort [4]. Dentally, oral mucosal infection, increased caries experience and alteration of the oral microbiome are commonly encountered [5,6]. The Sicca Symptoms Inventory (SSI) was developed as part of a comprehensive questionnaire tool, the Profile of Fatigue and Discomfort—Sicca Symptoms Inventory (PROFAD-SSI). The SSI can be used independently to assess the severity of sicca symptoms in SS and other rheumatic disorders [7,8].

The aim of this study was to assess the severity of self-reported sicca symptoms and investigate its relation to dental conditions, salivary flow rates and oral colonization of *Candida* of SS patients and non-SS individuals.

## 2. Materials and Methods

### 2.1. Study Design and Participants Recruitment

This was a cross-sectional case-control study. SS patients who fulfilled the American–European Consensus Group criteria [9] were recruited from rheumatology clinics of the Queen Mary Hospital (QMH) and Queen Elizabeth Hospital (QEH), Hong Kong. These two hospitals were selected because they were the two largest public hospitals in Hong Kong and are within 30 min travelling time to the Prince Philip Dental Hospital (PPDH). Potential recruits were excluded if they were less than 18 years of age, had received therapeutic irradiation to the head and neck region, had concurrent systemic illness (except connective tissue disorder associated with sSS) or were taking any medication that altered salivary flow. Healthy individuals attending the PPDH for routine follow-up who were age- and gender-matched to the first 40 SS subjects recruited were selected as controls. Socio-demographic information regarding education attainment, employment status and oral hygiene habits was recorded. A written informed consent was obtained from each participant. This study was approved by the Institutional Review Board of the University of Hong Kong/Hospital Authority Hong Kong West and Kowloon Central and East Clusters (UW 08-167 and KC/KE-10-0014/ER-2).

### 2.2. Assessment of Sicca Symptoms

SSI, in its original long form, contains 42 items covering four domains: ocular, oral, vaginal and cutaneous domains with one or more facets in each domain. In the short form version, sicca symptoms are grouped into 10 facets in the same four domains with three facets in the ocular domain, five facets in the oral domain and one facet each in the vaginal and cutaneous domains [7]. SSI short form was translated into Chinese and back translated into English by two dentists fluent in Chinese and English who were not involved in the study. Participants rated the severity of each symptom from “no problem at all” (score = 0) to “as bad as imaginable” (score = 7) over the last 2 weeks. A SSI summary score that ranged from 0 to 28 was computed by summing the mean score of each domain. The higher the score, the more severe the sicca symptoms.

### 2.3. Collection of Oral Rinse and Saliva

Procedures for oral rinse, unstimulated whole saliva (UWS) and stimulated whole saliva (SWS) collections and clinical examination have been described in detail in an earlier report [10]. In brief, all clinical procedures were carried out between 9 am to 12 noon. Oral rinse samples were collected by asking participants to rinse the mouth with deionized water for 30 s. After resting for 5 min and voiding the mouth, UWS was collected over 5 min where participants drooled or gently spat all saliva into a pre-weighed vial. SWS was collected by spitting all saliva into another sterile pre-weighed vial while chewing on a piece of silicone rubber tubing (2 mm × 8 mmØ) for 5 min. The pH of UWS and SWS was assessed at chairside using a pH meter (Sentron 501 Pocket FET pH meter, Sentron, WA, USA) immediately after saliva collection and the flow rates were determined and expressed as mL/min.

### 2.4. Dental Examination and Plaque Collection

Dental examination was carried out in a standard dental surgery room at PPDH using a mouth mirror and a WHO probe. Exposed tooth and root surfaces were dried with a 3-in-1 syringe. Dental plaque was collected from all accessible coronal tooth surfaces using a sterile sickle scaler and then dispersed into a pre-weighed vial containing 500 μL phosphate-buffered solution (PBS). Decayed (DS), missing (MS) and filled (FS) tooth surfaces (DMFS) were recorded according to the modified international Caries Detection and Assessment System (ICDAS) [11].

### 2.5. Identification of Candida in Dental Plaque and Oral Rinse

Oral rinse and dental plaque samples were transferred to the laboratory for microbial analysis immediately after collection as described in our earlier report [10]. Both samples were vortexed for 30 s at maximum setting and 1:10 diluted with 0.01 M sterile PBS (pH = 7.2). A 50-µL aliquot of the undiluted (oral rinse samples) and diluted samples (oral rinse and dental plaque samples) were spiral-plated onto the Sabouraud dextrose agar (SDA; Oxoid, Unipath Ltd., Basingstoke, Hampshire, UK) and CHROMagar (Becton Dickinson Europe, Le Pont de Claix, France) in duplicates and incubated at 37 °C in air for 48 h. Well-separated and evenly dispersed colonies on incubated plates were counted and subcultured on SDA plates to obtain pure isolates. The number of the *Candida* colonies was recorded and expressed as CFU/mL (oral rinse) or CFU/g (plaque). *Candida* identification was based on the colony morphology in CHROMagar and SDA, cell morphology, gram-stain reaction and result of the API ID 32C system (bioMérieux, Marcy l’Etoile, France).

### 2.6. Sample Size Requirement

The main study objective was to compare the prevalence of various self-reported sicca symptoms and oral conditions among the SS patients and non-SS patients. In order to detect a difference in proportions of at least 30% between the two groups at a statistical significance level of 0.05 and 80% power, a sample size of 39 people in each group was required.

### 2.7. Statistical Analysis

Socio-demographic data, SSI domain and summary scores and salivary, clinical and microbiological parameters were analyzed and compared among the pSS, sSS and control groups. Differences in categorical variables among the three groups were tested by Chi-squared tests or Fisher’s exact tests, whichever was appropriate. An unpaired *t*-test, one-way ANOVA or the Kruskal–Wallis H Test, whichever appropriate, was performed for evaluating differences in continuous variables among the three groups. Multiple comparisons were carried out with Bonferroni correction of *p*-values. Pearson’s correlation or Spearman’s rank correlation, whichever was appropriate, was used to explore associations between parameters of interest. The level of significance was set at 5% (*p* < 0.05). Data analysis was processed using SPSS software (IBM, Armonk, NY, USA).

## 3. Results

### 3.1. Characteristics of Participants

Eighty-five SS patients (mean age = 50.4 years, 2 men) and 40 healthy individuals (mean age = 51.4) participated in this study. The underlying secondary connective tissue diseases of sSS patients included systemic lupus erythematosus (SLE) (*n* = 26), rheumatoid arthritis (*n* = 14), scleroderma (*n* = 2), mixed connective tissue disorder (*n* = 2) and one each with Raynaud’s syndrome, seronegative arthritis and vasculitis. More than 70% of them had attained secondary education or above. About half of them were engaged in employment. The majority of the participants brushed their teeth twice or more per day with fluoride toothpaste and practiced interdental cleaning. However, only one-third of them used fluoride mouth rinse. No statistical significant difference in gender, education level, work status or oral hygiene practices was detected among the groups (Table 1).

SS patients had similar number of remaining teeth as compared to the non-SS individuals; however, the DMFS (*p* < 0.05) and FS (*p* < 0.001) scores were significantly higher (Table 1).

Mean SSI summary and all domain scores of SS patients were statistically significantly higher than the healthy controls (Table 1). SS patients had lower UWS and SWS flow rates than the controls (*p* < 0.001). The mean pH values of both UWS and SWS were within normal range.

### 3.2. Sicca Symptoms

Sicca symptoms were highly prevalent in SS patients (Table 2). Significantly more SS patients reported sicca symptoms in the oral, ocular and vaginal domains than the controls (*p* < 0.05). The most prevalent sicca symptoms were eye irritation (93%), followed by dry throat or nose (88%), need of fluid for mouth wetting (88%), dry or itchy skin (87%) and difficulty in eating (86%). In the control group, eye irritation (68%) and dry or itchy skin (63%) were reported by more than half of participants.

### 3.3. Oral Candida

The prevalence of oral *Candida* in the oral rinse and dental plaque of SS patients was 63.5% and 68.2%, respectively. Twenty-seven (27.1%) SS patients and all controls were free of oral *Candida*. *C. albicans* was the most frequently isolated *Candida* species, contributing to 52.9% and 57.6% of total *Candida* in the oral rinse and dental plaque samples, respectively. Mixed colonization of oral *Candida* was detected in 12.9% (oral rinse samples) and 11.8% (dental plaque samples) of patients. *C. dubliniensis* (7.2%) was the most frequent non-albicans species, followed by *C. tropicalis* (5.9%).

### 3.4. Correlations

SSI summary score and oral domain score were positively correlated to DMFS, FS and *Candida* counts in both oral rinse and dental plaque, and negatively correlated to UWS and SWS flow rates (Table 3). SSI summary score (*r_s_* = 0.30, *p* = 0.006) and cutaneous domain score (*r_s_* = 0.44, *p* < 0.001) were positively correlated to the time since disease diagnosis in SS patients. *Candida* counts were negatively correlated to salivary flow rates and positively correlated to DMFS, DS and FS (Table 4).

## 4. Discussion

Sicca symptoms are typical complaints of SS patients. A number of subjective measures have been developed to capture different sicca features. For instance, the Xerostomia Inventory (XI) [12] and the Ocular Surface Disease Severity Index (OSDI) [13] are designed to assess oral and ocular dryness, respectively. SSI is a disease-specific questionnaire developed based on patient interviews and published sicca symptom questions, and has been validated in terms of face and construct validity and reliability. It is mainly used as a screening tool in SS and other rheumatic disorders but has also been suggested useful as an outcome tool [7,8].

In general, the prevalence of sicca symptoms is higher in the ocular and oral domains than that of the vaginal and cutaneous domains. This finding echoes other reports that dryness of the eyes and mouth are the predominant complaints of patients with SS. In a group of 169 SS patients, 93.5%, 67.5%, 38.5% and 55.6% complained of dry mouth, dry eyes, dry vagina and dry skin, respectively [14]. The same study also found that dry mouth was the best predictor symptom of SS. In fact, it has been reported that presence of oral symptoms preceded the diagnosis of SS by 8 years [15]. In this study, eye irritation (93%) was the most prevalent sicca symptom among all sicca symptoms investigated, which is in concordance with reports on some SS patient groups [16], while other studies reported a higher prevalence of oral symptoms than symptoms of other regions [14]. Nonetheless, this finding may not be surprising as ocular problems e.g., dry eyes and eye irritation due to SS, were reported to be more severe in cities with air pollution, and Hong Kong is a metropolitan city with a moderately high degree of air pollution [17].

SS patients had higher mean SSI summary and domain scores in all four domains than the controls. Nonetheless, the summary and individual domain scores were all below midpoint of the scale which implies that SS patients in this study perceived their sicca symptoms to be moderately severe. SS patients have been described as relatively stoical for both ocular and oral dryness in a study that reported that more than 80% of them had self-reported dryness at a relatively low level compared to objective findings [18]. Furthermore, results of this study also indicated that sicca symptoms in ocular, oral, vaginal and cutaneous domains due to SS can be discriminated by the SSI. It is also noteworthy that SSI scores between pSS and sSS patients had no statistically significant difference, which mirrored the findings of a previous report [7]. This implies that the severity of sicca symptoms is similar between primary and secondary SS patients.

Severity of cutaneous manifestations of SS is said to be underestimated mainly due to its less pronounced subjective manifestation and objective consequences than oral and ocular dryness [19]. Xeroderma, the most common skin manifestation of SS, is believed to be secondary to a disruption of the biochemical properties of the epidermis and is frequently complicated by pruritus and burning symptoms [20]. Although SSI used “painful sex” as a facet of the vaginal sicca, it was reported that more than 70% of SS patients presented with gynecological symptoms, leading to impaired sexual intercourse [21]. Another report found that premenopausal women with SS were less satisfied with sexual activity due to vaginal dryness, physical pain and impaired function due to the disease [22].

Correlation between the objective measures of dryness and subjective sicca symptoms has been reported to be poor in the general population [23]. However, the negative correlations between the SSI summary scores, the ocular and oral domain scores, and the UWS and SWS flow rates obtained in this study were in concordance with another study that reported a negative correlation between oral dryness and USW flow [18]. Unstimulated salivary flow was reported to be negatively correlated with difficult eating, dry throat and oral problems in pSS patients, and to difficult eating and wetting mouth in sSS patients with systemic lupus erythematous [7]. SS patients in this study have long-standing diseases, with an average time since diagnosis of 8.5 years. Damage to the exocrine tissues, especially the salivary and the lacrimal glands, is substantial. The preciseness of SSI in its severity scores of grouped symptoms and the frequencies of single symptoms of sicca allows reflection of the true association between subjective complaints and objective measurement of hyposalivation.

In addition, duration of SS exhibited weak and moderate correlations to SSI summary scores (*r* = 0.30, *p* = 0.007) and cutaneous domain scores (*r* = 0.45, *p* < 0.001), respectively, but not to other domain scores. Patients suffering from long-standing chronic illnesses are likely to have developed some coping strategies. For instance, SS patients tend to avoid dry and spicy food, as dry food is difficult to swallow and causes choking while spicy food induces mucosal pain. SS patients also put on protective eyewear and use humidifiers in dry weather. However, dry skin and chronic itch can be very disturbing, in particular when many parts of the body are affected. It has been reported that pSS patients with chronic itch reported a low quality of life and up to 80% had sleep disturbances [24]. The pathogenesis of itch in SS in still unclear and a specific treatment has yet to be developed [25].

Caries experience of SS patients in this study appears less pronounced than that reported in other studies where the mean DMFS ranged between 83 and 106.1 [26,27,28]. This difference may be explained by the fact that SS patients of this study were about 10 years younger than those reported in other studies. In addition, water fluoridation has been implemented in Hong Kong for over 50 years that provides caries protection in a passive manner. Nonetheless, the F-component of DMFS exhibited significant positive correlations with SSI summary, ocular and oral domain scores. This correlation implies that patients who presented with high caries experience may warrant an investigation for the presence of concurrent ocular and oral sicca symptoms, and vice versa. Healthcare workers should pay attention to these signs and symptoms, so that referrals can be made for early diagnosis, prompt treatment and effective management of the symptoms. The participants in this study were regular dental attendees. Dental problems such as dental caries were promptly treated, especially for the SS patients, as they were well informed of their high caries risk. The number of filled tooth surfaces reflects the damage SS has caused. Despite having good oral hygiene practices, the use of additional fluoride apart from fluoridated toothpaste is low. Daily high fluoride toothpaste and fluoride-containing oral gel, as well as chlorhexidine mouthwash, have been recommended for caries control in SS patients [29].

Another interesting finding is the negative correlation between *Candida* counts and salivary flow rates. This finding echoes other studies that reported positive correlations between *Candida* counts and DMFS, DS and FS scores [30,31]. Similar correlations have been reported in some experimental [32] and epidemiological studies [33,34,35,36]. A recent meta-analysis reports that children with oral *C. albicans* have more than 5 times higher odds of having early childhood caries than those without *C. albicans* [37]. The role of *Candida* in the pathogenesis of caries warrants further investigations.

To our knowledge, this study was the first to obtain data of sicca symptoms in southern Chinese SS patients using SSI, and to explore correlations between SSI scores and salivary flow, caries experience and oral *Candida* colonization. The major limitation of this study was the relatively small number of participants. The comparison group was regular dental attendees to a dental hospital whose oral health condition may not be generalizable to that of the general public.

## 5. Conclusions

SS patients had more severe sicca symptoms than healthy individuals and the severity of self-reported sicca symptoms was negatively correlated to the salivary flow rates, but positively correlated to the caries experience and oral *Candida* colonization.

## Figures and Tables

**Table 1:** Socio-demographic profile, oral hygiene practice, Sicca Symptoms Inventory (SSI) scores, salivary profile and dental condition by group.

Parameters under Investigation	pSS (*n* = 38)	sSS (*n* = 47)	Control (*n* = 40)	*p*-Value
**Sociodemographic**				
Mean age	50.8 ± 8.5 (35–71)	50 ± 10.5 (26–75)	51.4 ± 7.4 (37–98)	n.s.
Number of female	36 (94.7%)	47 (100.0%)	40 (100.0%)	n.s.
Mean time since diagnosis (months)	82.8 ± 54.1 (2–240)	118.1 ± 86.9 (2–425)	not applicable	n.s.
Education level primary school or below secondary school or above	23.7% 76.3%	29.8% 70.2%	32.5% 67.5%	n.s.
Employed	57.9%	48.9%	45.0%	n.s.
**Oral hygiene practices**				
Brushed twice or more daily Practiced interdental cleaning Used fluoride toothpaste daily Used fluoride mouth rinse daily	94.7% 71.1% 89.5% 34.2%	93.6% 72.3% 97.9% 34.0%	92.5% 60.0% 95.0% 20.0%	n.s. n.s. n.s. n.s.
**SSI**				
Mean SSI summary score	10.8 ± 4.9 (3.0–21.1)	11.4 ± 5.4 (1.5–22.5)	5.4 ± 4.8 (0–21.1)	<0.001 *
Mean ocular domain score	2.8 ± 1.6 (0–6.7)	2.9 ± 1.7 (0–6.3)	1.5 ± 1.4 (0–5.3)	<0.001 *
Mean oral domain score	2.7 ± 1.5 (0–5.4)	2.6 ± 1.6 (0.2 –6.4)	0.7 ± 0.9 (0–3.4)	<0.001 *
Mean vaginal domain score ^#^	2.3 ± 2.2 (0–7.0)	2.5 ± 2.2 (0–7.0)	1.4 ± 2.1 (0–7.0)	0.023 *
Mean cutaneous domain score	3.0 ± 1.9 (0–7.0)	3.4 ± 2.0 (0–7.0)	1.9 ± 1.9 (0–7.0)	0.001 *
**Salivary profile**				
Mean UWS flow rate (mL/min) Mean UWS pH ^§^	0.2 ± 0.2 (0–1.0) 7.2 ± 0.4 (6.5–8.5)	0.3 ± 0.2 (0–1.1) 7.4 ± 0.5 (6.3–8.3)	0.5 ± 0.3 (0.1–1.3) 7.6 ± 0.4 (6.8–8.3)	<0.001 * <0.001 *
Mean SWS flow rate (mL/min) Mean SWS pH ^¶^	0.4 ± 0.5 (0–2.0) 7.7 ± 0.4 (6.8–8.5)	0.5 ± 0.5 (0–2.6) 7.8 ± 0.5 (6.5–6)	1.1 ± 0.6 (0.2–2.5) 7.8 ± 0.3 (7.2–8.2)	<0.001 * n.s.
**Dental condition**				
Mean number of teeth	26.2 ± 4.0 (12–2)	27.2 ± 3.1, (18–32)	26.1 ± 3.7 (14–32)	n.s.
Mean DMFS	46.5 ± 24.7 (4114)	39.9 ± 19.1 (1–75)	33.1 ± 19.6 (5–90)	0.022 *
Mean DS	1.2 ± 1.3 (0–4)	0.7 ± 0.9 (0–3)	1.1 ± 2.5 (0–13)	n.s.
Mean MS	27.3 ± 19.8 (0–94)	23.0 ± 14.4 (0–65)	25.6 ± 18.4 (0–90)	n.s.
Mean FS	18.0 ± 9.9 (0–43)	16.1 ± 12.7 (0–54)	6.4 ± 5.4 (0–22)	<0.001 *

pSS: primary Sjögren’s syndrome; sSS: secondary Sjögren’s syndrome; n.s.: not significant; SSI: Sicca Symptoms Inventory; UWS: unstimulated whole saliva; SWS: stimulated whole saliva; DMFS: decayed, missing, filled surface; DS: decayed surface; MS: missing surface; FS: filled surface. ^#^ Only female participants responded in this domain. ^§^ 4 pSS and 19 sSS patients failed to produce any measurable amount of UWS. ^¶^ 1 pSS and 11 sSS patients failed to produce any measurable amount of SWS. * Significant difference between pSS and sSS vs. control (Kruskal–Wallis test).

**Table 2:** Prevalence (%) of sicca symptoms.

Domain	Facet	pSS (*n* = 38)	sSS (*n* = 47)	Controls (*n* = 40)
Ocular	sore eyes **	84.5	85.1	37.5
	eye irritation **	94.7	91.5	67.5
	poor vision **	63.2	80.9	45.0
Oral	difficulty eating **	92.1	80.9	12.5
	dry throat or nose **	89.5	87.2	32.5
	bad breath *	73.7	74.5	50.0
	wetting mouth **	86.8	87.2	25.0
	other oral problems **	63.2	57.4	25.0
Vaginal ^¶^	painful sex **	70.3	69.6	37.5
Cutaneous	dry or itchy skin *	89.5	85.1	62.5

pSS: primary Sjögren’s syndrome; sSS: secondary Sjögren’s syndrome. * *p* < 0.05 (Chi-squared test). ** *p* < 0.01 (Chi-squared test). ^¶^: male patient from each SS group was excluded from analysis.

**Table 3:** Correlations (r_s_ values) between SSI scores and dental conditions, salivary flow rates and *Candida* counts.

SSI Summary and Domain Scores	DS	MS	FS	DMFS	UWS Flow Rate	SWS Flow Rate	*Candida* Counts in Oral Rinse	*Candida* Counts in Dental Plaque
SSI summary score	0.07	0.01	0.26 **	0.20 *	−0.26 **	−0.36 **	0.28 **	0.28 **
Ocular domain score	0.03	−0.12	0.18 *	0.03	−0.26 **	−0.28 **	0.25 *	0.24 *
Oral domain score	0.13	−0.01	0.40 **	0.25 *	−0.40 **	−0.44 **	0.42 **	0.49 **
Vaginal domain score	−0.08	0.02	0.07	0.11	−0.10	−0.20 *	0.10	0.08
Cutaneous domain score	0.08	0.12	0.21 *	0.21 *	−0.048	−0.18	0.16	0.15

SSI: Sicca Symptoms Inventory; DS: decayed surface; MS: missing surface; FS: filled surface; DMFS: decayed, missing, filled surface; UWS: unstimulated whole saliva; SWS: stimulated whole saliva. * *p* < 0.05, Spearman’s correlation ** *p* < 0.005, Spearman’s correlation.

**Table 4:** Correlations (r_s_ values) between *Candida* counts and salivary flow and dental conditions.

Salivary Flow Rates and Dental Conditions	*Candida* Counts	
	in Oral Rinse	in Dental Plaque
UWS flow rate	−0.70 **	−0.71 **
SWS flow rate	−0.66 **	−0.65 **
DMFS	0.52 **	0.44 **
DS	0.47 **	0.42 **
MS	0.24 *	0.17
FS	0.51 **	0.48 **

UWS: unstimulated whole saliva; SWS: stimulated whole saliva; DMFS: decayed, missing, filled surface; DS: decayed surface; MS: missing surface; FS: filled surface. * *p* < 0.05, Spearman’s correlation ** *p* < 0.005, Spearman’s correlation.
